# Detecting responses to treatment with fenofibrate in pedigrees

**DOI:** 10.1186/s12863-018-0652-5

**Published:** 2018-09-17

**Authors:** Svetlana Cherlin, Maggie Haitian Wang, Heike Bickeböller, Rita M. Cantor

**Affiliations:** 1 Institute of Genetic Medicine, International Centre for Life, Newcastle University, Central Parkway, Newcastle upon Tyne, NE1 3BZ UK; 2 Division of Biostatistics, Centre for Clinical Research and Biostatistics, JC School of Public Health and Primary Care, The Chinese University of Hong Kong, Shatin, N.T., Hong Kong SAR, China; 3 Department of Genetic Epidemiology, University Medical Center, Georg-August University Göttingen, Humboldtallee 32, 37073 Göttingen, Germany; 4 Department of Human Genetics, David Geffen School of Medicine at UCLA, 695 Charles E. Young Dr. South, Los Angeles, CA 90095 USA

**Keywords:** Fenofibrate treatment, GOLDN study, Triglycerides, Epigenetics, Predictive modeling

## Abstract

**Background:**

Fenofibrate (Fb) is a known treatment for elevated triglyceride (TG) levels. The Genetics of Lipid Lowering Drugs and Diet Network (GOLDN) study was designed to investigate potential contributors to the effects of Fb on TG levels. Here, we summarize the analyses of 8 papers whose authors had access to the GOLDN data and were grouped together because they pursued investigations into Fb treatment responses as part of GAW20. These papers report explorations of a variety of genetics, epigenetics, and study design questions. Data regarding treatment with 160 mg of micronized Fb per day for 3 weeks included pretreatment and posttreatment TG and methylation levels (ML) at approximately 450,000 epigenetic markers (cytosine-phosphate-guanine [CpG] sites). In addition, approximately 1 million single-nucleotide polymorphisms (SNPs) were genotyped or imputed in each of the study participants, drawn from 188 pedigrees.

**Results:**

The analyses of a variety of subsets of the GOLDN data used a number of analytic approaches such as linear mixed models, a kernel score test, penalized regression, and artificial neural networks.

**Conclusions:**

Results indicate that (a) CpG ML are responsive to Fb; (b) CpG ML should be included in models predicting the TG level responses to Fb; (c) common and rare variants are associated with TG responses to Fb; (d) the interactions of common variants and CpG ML should be included in models predicting the TG response; and (e) sample size is a critical factor in the successful construction of predictive models representing the response to Fb.

## Background

The Genetics of Lipid Lowering Drugs and Diet Network (GOLDN) study was designed to investigate potential contributors to the effects of fenofibrate (Fb) on triglyceride (TG) levels [1]. Because of the variety of genetic, genomic, and trait measures, the longitudinal nature of the data encompassing a drug treatment, and the challenges provided by the nonindependence of family members, this rich GAW20 data set led to a broad spectrum of analyses. In addition to the real GOLDN data, simulated data provided the opportunity to formally evaluate statistical approaches. Here we summarize the research questions, study designs, and analytic approaches used by the members of the Genetics of Treatment Response group of 8 papers focused on association and prediction analyses of treatment response. We provide a brief summary of their findings; for a more complete understanding of these investigations, we suggest reading the published manuscripts [2–9].

Fb is a treatment for elevated TG levels. There are well-known outcomes, although individual responses are highly heterogeneous. Binding of Fb activates peroxisome proliferator-activated receptor-α, starting a cascade that leads to the modulation of genes that regulate lipoprotein metabolism and inflammation. Reduced levels of lipids and lipoproteins, including TGs, are observed after fasting, and even stronger effects are observed after a fatty meal. There are also many nonlipid effects, such as improvements in biomarkers of inflammation. Treatment is often administered to individuals with high lipid levels when changes to diet and exercise either cannot be followed or do not show the desired results [10]. There are many genes involved in the variation in lipids and inflammation, and DNA methylation may play a central role in baseline TG levels, as well as in posttreatment TG levels, by affecting chromatin structure and altering the availability of coding regions in the transcription process [11]. In the GOLDN study, treatment with micronized Fb consisted of 160 mg per day for 3 weeks.

A number of well-established statistical approaches are available for modeling and/or predicting the treatment response, depending on the goals of the study. These include full longitudinal data analysis, pretreatment and posttreatment analysis, summary statistics, and end-point analyses, including survival analysis. For longitudinal analyses we use full information of the course of the considered outcome, and for the others we can use aggregated or partial information. Often linear mixed models (LMMs) or generalized estimating equations (GEEs) are incorporated to address the correlations within the data. There are several ways regression models can be applied to analyze pretreatment and posttreatment data. Three common models are (a) a follow-up model, (b) a change analysis, and (c) an analysis of covariance (ANCOVA) model. They are:

- Follow-up: postTG = a + b × covariates + error
- Change: postTG − preTG = a + b × covariates + error
- ANCOVA: postTG = a + b × covariates + c × preTG + error

where *postTG* is posttreatment TG level and *preTG* is pretreatment TG level. The definition of *Change* can be based either on a difference or a ratio, depending on whether a log-transformation is applied. A *Percent Change* model can also be applied, where an appropriate value is between 30 and 50%, depending on ln *preTG* [10]. A binary cutoff such as 30% change, indicating that the responders and nonresponders to treatment can also be applied. An example of a summary analysis is provided by Irvin et al. [12], who used these same GOLDN data to consider the area under the curve of TG at several points in time before and after the high-fat meal.

The focus of most of the 8 manuscripts in this group was predicting the TG level response to Fb treatment. There were several complexities in these data, including the nonindependence of subjects in the pedigrees and the large number of single-nucleotide polymorphism (SNP) markers, genome-wide. The analytic approaches were fairly broad, and the most common was LMM, which tests for associations between the markers and the trait, while adjusting for the statistical nonindependence of members of the same pedigree. An alternative approach, the kernel score test (KST), used by Yasmeen et al. [9] allows one to test a set of markers for overall association with a trait, using a semiparametric procedure. LASSO (least absolute shrinkage and selection operator) regression used by Cherlin et al. [3] predicts the treatment response from markers based on shrinking regression coefficient toward zero through a penalty on the coefficients. Xia et al. [6] applied an artificial neural network (ANN), a semiparametric modeling technique that does not require linearity.

TG and methylation levels (ML) at approximately 450,000 epigenetic markers were measured pretreatment and posttreatment in the study participants, allowing a wide variety to the analyses. In addition, approximately 1 million SNPs were genotyped or imputed in each of the study participants drawn from 188 pedigrees, permitting the integration of clinical, genetic, and genomic data. Each paper used the SNPs to inform their analyses, however only some used the TG data [3, 4, 6–9] or epigenetic data [2, 5, 6, 9]. Most used the full family data, while one paper selected independent individuals from each pedigree [9], and another investigated the impact of approaches to correcting for family structure [4].

Two contributions analyzed the simulated data [3, 9]. Both knew the answers prior to the analysis and both used the suggested simulated replicate (84). The GAW20 simulated data used the same family structure, genotypes, and pretreatment TG and ML as the real data, with posttreatment TG levels simulated using a model with 5 causal SNPs influencing posttreatment TG levels fully, only when they were unmethylated. These cytosine-phosphate-guanine (CpG) markers, along with another 5, increased posttreatment TG level variability.

Although SNP and TG level data were analyzed to identify predictors of TG level response by most of the investigators, the inclusion of longitudinal epigenetic and TG level data pretreatment and posttreatment provided an opportunity for interesting research questions, along with some analytic challenges, regarding ML under drug treatment. Research on epigenetic responses to Fb or other drugs has not been extensive, and the GOLDN study is exceptional in providing these longitudinal treatment response data. ML are highly variable and depend on factors such as age, smoking, treatment, and laboratory/batch/probe effects and cell type used. Here, they were assayed from the CD4+ T cells extracted from whole blood. Because of the widespread effects of lipids and inflammation, it is hypothesized that this cell type should be sufficient in the GOLDN study.

Beta scores, which correspond to the methylated proportion of total signal from the population-specific probe, were provided. Two different probes were used for pretreatment and posttreatment, which had serious implications, producing a confounding batch effect that presented analytic challenges for detecting Fb-responsive CpG sites [2] and interpreting methylation quantitative trait loci (meQTL) analyses [5]. To account for the batch effect, Cantor et al. [2] compared the ranks of posttreatment and pretreatment ML familial and variability distributions to detect those CpG sites exhibiting a likely genetic change in response to treatment with Fb. This was followed by a meQTL analysis of a filtered number of candidate responsive CpG sites. Wu et al. [5] conducted a full genome *cis*-meQTL analysis identifying many more such sites, while accounting for 2 different probes.

The most common considerations of all contributions were adjusting for family structure and use of covariates in the analyses. LMM were used by most to adjust for family structure [2, 5–7]. Other approaches included principal components (PCs) [3] or GEE [6], while Yasmeen et al. [9] included only unrelated individuals in the analysis. Hsu et al. [4] investigated different approaches of adjusting for family structure using kinship matrices. In most analyses, the covariates included nongenetic effects such as age, sex, center, smoking status, and a variable number of PCs.

In the subsequent sections, we provide greater detail regarding (a) the primary research questions posed by the 8 studies; (b) the analytic approaches used to investigate them; (c) the data used in the analyses; (d) the primary findings of each study; (e) a discussion of the factors that help us interpret the primary findings; and (f) the conclusions that can be drawn from this collective work. Greater detail on the individual studies can be gleaned from the original manuscripts [2–9].

## Methods

### GOLDN study design

The GOLDN study used a longitudinal design [13, 14] to study the TG level response to Fb. Participants were middle-aged, self-reported white individuals, likely to be genetically homogenous, who were recruited through a previous family study [12] from 2 centers in the United States. Individuals with extreme TG measures and/or a recent history of severe cardiovascular disease were excluded. Participants were asked to fast and abstain from alcohol, and not take lipid-lowering drugs for at least 4 weeks prior to the inception of the study. A high-fat meal challenge was given twice, 3 weeks apart, during which time Fb treatment was given to lower lipids.

Lipid levels, including TGs, were measured approximately 1 day prior to the drug intervention, with times 1 and 2 corresponding to measurements taken prior to treatment and times 3 and 4 corresponding to measurements taken after the treatment, within each pair (TG1 and TG2, TG3 and TG4) only 1 or 2 days apart. One can assume that TG1 and TG2 as well as TG3 and TG4 measure the same levels, except for random variation. The 8 papers in this group focused on treatment effects by using either T2 or T4, or the means of T1 and T2 or T3 and T4. These are denoted as *preTG* and *postTG*, respectively. Because TG levels have a skewed distribution, some authors used log-transformed triglyceride (lnTG).

Genetic markers were array-based SNPs. Epigenetic marker ML were beta-scores of CpG markers using a methylation array, based on CD4+ T cells and measured only at time points 2 and 4. The GAW20 simulations used the same data, except that posttreatment TG levels were simulated based on linear models.

### Analytic approaches used in the 8 genetics of treatment response contributions

Our Genetics of Treatment Response groups investigated associations with and predictions of responses to Fb. There is a marked distinction in the aims of association and prediction analyses, although both can begin with the same CpG ML and TG levels data in a single study sample. Association analysis tests each CpG ML to identify those having a significant relationship with TG levels in the population under analysis. The goal is to then use bioinformatics and functional studies to reveal the biology driving these associations. Prediction analysis focuses on using the data in the study sample to develop an analytic model composed of CpG ML in order to predict TG levels. The ultimate goal is to use this prediction model and CpG ML in individuals who have not had their TG levels measured for prediction of their specific TG level. As an example, regression models are widely used for prediction.

Table 1 summarizes the aims of each project and the analytic methods used, in alphabetical order of the first named author. While most contributions concentrated on SNPs and ML as predictors in association analyses [2, 5–7, 9], Yang and Chen [8] used a homozygosity intensity measure. Contributions that investigated the association between the SNPs and the TG treatment response used the full genome-wide data [3, 4, 6–9]. Wu et al. [5] searched for *cis*-meQTL around each CpG site, genome-wide. Some did more targeted analyses. Cantor et al. [2] tested specific CpG sites showing a likely genetic response to treatment for meQTL. Yasmeen et al. [9] tested TG with genomic regions chosen around causal and noncausal CpG sites, based on the simulated data. Xia et al. [6] tested a subset of SNPs chosen by GEEs. Most contributions used LMMs to perform association tests [2, 5–7], whereas Yasmeen et al. [9] applied KST and linear regression. Prediction analyses employed penalized regression [3] and ANNs [6] with 10-fold cross validation. Below, we provide details regarding the primary analytic approaches used by the 8 papers in our group.

**Table 1:** Primary aims and statistical modeling methods

First Named Author	Aims of the Analysis	Analytic Methods
Cantor	Filter CpG sites for those exhibiting genetic contributions to ML; targeted meQTL studies	Concordance of familiality and variability of CpG distributional outliers, LMM
Cherlin	Predicting TG response to Fb with SNPs	LASSO penalized regression
Hsu	Evaluating adjustments for family structure	LMM
Wu	Genome-wide *cis*-meQTL studies	LMM
Xia	Evaluate ML in predicting TG response to Fb	ANN, GEE, and LMM
Xu	Predicting TG response to Fb with SNPs	LMM and KST
Yang	Association between homozygosity intensity and TG response to Fb	GEE
Yasmeen	Predicting TG response to Fb with SNPs and CpG ML	KST and linear regression

*ANN* Artificial neural networks, *CpG* Cytosine-phosphate-guanine, *Fb* Fenofibrate, *GEE* Generalized estimating equations, *KST* Kernel score test, *LASSO* Least absolute shrinkage and selection operator, *LMM* Linear mixed models, *meQTL* Methylation quantitative trait locus, *ML* Methylation level, *SNPs* Single nucleotide polymorphisms, *TG* Triglyceride levels

#### Linear mixed models

LMM approaches are widely used in genetic studies of pedigrees. This method assumes that the expected value of a trait is a linear combination of fixed and random effect predictors. Although genetic and covariate effects are modeled as fixed, family effects are considered random effects. LMM can be described as follows:

$$ \boldsymbol{y}=\boldsymbol{X}{\boldsymbol{\beta}}^{\boldsymbol{T}}+\boldsymbol{u}+\boldsymbol{\epsilon} $$

where ***y*** is a vector of traits; ***X*** is the SNP data coded according to the minor allele count; ***β*** is a vector of the regression coefficients for fixed effects; ***u*** is a vector of random effects, $\mathbf{u}\sim N\ \left(\mathbf{0},2{\sigma}_g^2\boldsymbol{\varPhi} \right)$, in which ***Φ*** is a matrix of pairwise kinship coefficients; and $\boldsymbol{\epsilon} \sim N\ \left(\mathbf{0},{\sigma}_{\epsilon}^2\boldsymbol{I}\right)$ is a vector of the residuals (***I*** is the identity matrix). The covariance matrix $2{\sigma}_g^2\boldsymbol{\varPhi}$ is block-diagonal with 1 block per family. The kinship matrix can be calculated from a known pedigree structure, or from the genetic data when the pedigree structure is not available. Different implementations allow the kinship matrix to be estimated separately from the association testing, thus allowing for the use of alternative packages for kinship matrix estimation when performing association tests. Kinship matrices calculated using different methods tend to differ from each other. There is little difference, however, between the results of the association or prediction analyses obtained using different estimation methods [15].

#### Kernel score test

In KST, the trait ***y*** is expressed as:

$$ \boldsymbol{y}=\boldsymbol{X}{\boldsymbol{\beta}}^{\boldsymbol{T}}+h\left(\boldsymbol{Z}\right)+\boldsymbol{\epsilon} $$

where ***X*** is a matrix of known fixed covariates; ***β*** is a vector of the regression coefficients; $\boldsymbol{\epsilon} \sim N\ \left(\mathbf{0},{\sigma}_{\epsilon}^2\boldsymbol{I}\right)$ is a vector of residuals; *h*(***Z***) ***= Ka***^*T*^ is a nonparametric function that depends on the kernel matrix ***K = ZZ***^*T*^; and ***a*** is a vector of random effects, ***a***~*N*(**0**, *τ****K***). The matrix ***Z*** is the matrix of the markers, and *τ* is the genetic covariance component. KST investigates whether the genetic covariance component equals zero (ie, *τ* = 0) [16], which can be interpreted as of test of whether there are aggregated genetic effects contributing to the trait, ***y***.

#### Least absolute shrinkage and selection operator

LASSO [17] is a penalized regression model.

The trait ***y*** is expressed as:

$$ \boldsymbol{y}=\boldsymbol{X}{\boldsymbol{\beta}}^{\boldsymbol{T}}+\boldsymbol{\epsilon} $$

where ***X*** is a matrix of known fixed covariates; ***β*** is a vector of the regression coefficients; and $\boldsymbol{\epsilon} \sim N\ \left(\mathbf{0},{\sigma}_{\epsilon}^2\boldsymbol{I}\right)$ is a vector of residuals. LASSO allows shrinkage of the estimators of the regression coefficients in a linear model toward zero using a penalty. The estimators of the regression coefficients ***β*** are found by minimising the sum of the residual sum of squares and a penalty function:

$$ {\widehat{\beta}}_o,\widehat{\boldsymbol{\beta}}=\mathrm{argmin}\ \left[\sum \limits_{i=1}^n{\left({y}_i-{\beta}_o-\sum \limits_{j=1}^p{\beta}_j{x}_{ij}\right)}^2+\lambda \left\Vert \boldsymbol{\beta} \right\Vert {}_{\ell_1}\right] $$

where *λ* is a regularization parameter that controls the amount of shrinkage, and $\left\Vert \boldsymbol{\beta} \right\Vert {}_{\ell_1}$ is an *ℓ*_1_-norm penalty which is a sum of the absolute values of the coefficients (ie, $\left\Vert \boldsymbol{\beta} \right\Vert {}_{\ell_1}$ = $\sum \limits_{j=1}^p\mid {\beta}_j\mid$ [*p* is a number of markers]). One important property of the LASSO penalty is that it allows the coefficients to be set to exactly zero, thus performing variable selection.

#### Artificial neural network

ANN is a computational model based on a collection of nodes that are connected in layers, where the signal travels from the input layer to the output layer, including possible hidden layers. An ANN consists of the interconnections between different layers of nodes, the weights of the interconnections, and of the activation function for converting a node’s weighed input to its output.

Each layer of an ANN can be described by a neural network function as follows:

$$ {f}_i\left({x}_i\right)=g\left(\sum \limits_j{w}_{ij}{x}_j+{b}_j\right) $$

where index *i* represents the nodes of a layer; index *j* represents input nodes; *w*_*ij*_ and *b*_*j*_ are weights; and *g* is an activation function [18]. Different layers can employ different activation functions. In our group, Xia et al. [6] used a 3-layer ANN with a hyperbolic tangent sigmoid transfer function as an activation function for the hidden layer, and a linear function as an activation function for the output layer. Different algorithms are available for training an ANN. For example, Xia et al. [6] used an adaptive gradient descent with momentum as a training method for ANN.

### Study designs used in the genetics of treatment response contributions

Table 2 summarizes the study designs employed by our 8 GAW20 contributions, in alphabetical order of the first named author. The second column gives the outcomes assessed for the treatment response of that study. For example, in row 1, Cantor used the top 0.1% of the ranks of posttreatment ML sibling correlations (sib corrs) and SDs to select the CpG sites likely to be responsive to treatment, followed by a meQTL analysis, while in row 2, Cherlin used the log of the posttreatment TG levels as the predicted treatment response. The third column indicates that both studies used SNPs as predictors, and the fourth column indicates they used pretreatment ML and pretreatment TG levels as baseline measures, respectively. The next three columns indicate whether PCs were used in the analysis, and if so, how many, the covariates used, and how the study addressed family structure. Most contributions adjusted for some covariates as well as between 10 and 20 PCs for SNPs or 4 PCs for ML, and adjusted for kinship via random effects in the model. One contribution investigated sibling pairs to identify likely heritable CpG ML [2], one used only independent individuals [9], and Hsu et al. [4] evaluated the approaches for adjusting for nonindependence of family members, including analyzing independent individuals.

**Table 2:** Design elements of studies addressing fenofibrate treatment effects

First Named Author	Outcome Variable	Genetic & Genomic Predictors	Baseline Measures	PCs	Covariates Included	Treatment of Family Data
Cantor	Post ML sib corrs & SDs, meQTL	SNPs	Pre ML sib corrs & SDs			LMM
Cherlin	Ln (postTG)	SNPs	Ln (preTG)	20	Age, center, smoking	PCs
Hsu	PreTG	SNPs		4	Age, sex, center	LMM Independents
Wu	Ln (postML − preML)	SNPs		10	Age, sex, batch, smoking	LMM
Xia	(PostTG − preTG)/preTG Binary TG		PreTG	10	Age, sex, smoking, ML	Empirical kinships
Xu	PostTG − preTG	SNPs		10	Age, center, ATP, smoking, IDF	LMM
Yang	PostTG − preTG	SNPs		10	Age, sex, center, ATP, smoking, IDF	GEE
Yasmeen	Ln (postTG)	ML, SNPs	Ln (preTG)	none	Age	Independents

*ATP* Adult Treatment Panel, *IDF* International Diabetes Federation, *LMM* linear mixed model, *Ln* natural logarithm, *meQTL* methylation quantitative trait locus, *ML* Methylation levels, *PCs* Number of principal components; pre, pretreatment; post, posttreatment, *SDs* Standard deviations; sib corrs, sibling correlations, *SNPs* Single-nucleotide polymorphisms, *TG* Triglyceride levels

## Results

The investigations we report here are focused on the response to treatment with Fb. However, the study designs and analytic approaches used are quite varied, and the results are fairly broad. Table 3 presents the main results of the studies in alphabetical order of the first named author.

**Table 3:** Primary results for GAW20 treatment response group

First Named Author	Results
Cantor	Genetic screening of ML identifies *ANAPC2* and *KIAA1804* as Fb responsive*;* rs3087779 and rs1294198 are meQTL for those genes.
Cherlin	LASSO regression on LD-pruned GWAS data provides low prediction power in simulated and real data; increasing samples to 7 K provides detectable signals and reasonable prediction accuracy.
Hsu	LMM is the preferable approach when adjusting for family structure.
Wu	Genome-wide studies identify 229 *cis*-meQTL for 610 CpG sites using LMM; rs3733749 and cg00514575, upstream of *MGAT1* is strongest signal.
Xia	Adding CpG ML to a neural network with SNPs and clinical traits improves prediction of TG response to Fb by 4%.
Xu	TG LMM identifies 4 significant SNPs, including rs964184 previously associated with lipid lowering statins.
Yang	*MACROD2* homozygosity intensity is associated with the TG response to Fb using genome-wide GEE*.*
Yasmeen	Including CpG–SNP interactions improves a KST TG prediction model; previously reported *CPT1A* is nominally replicated.

*CpG* Cytosine-phosphate-guanine, *Fb* Fenofibrate, *GEE* Generalized estimating equation, *GWAS* Genome-wide association study, *KST* Kernel score test, *LASSO* Least absolute shrinkage and selection operator, *LD* Linkage disequilibrium, *LMM* Linear mixed models, *meQTL* Methylation quantitative trait locus, *ML* Methylation level, *SNP* Single-nucleotide polymorphism, *TG* Triglyceride

### Support for CpG responses to treatment with fb

Four studies [2, 5, 6, 9] addressed the role of CpG sites in response to Fb. Two provided support for a CpG ML response to treatment with Fb. A third showed the importance of CpG ML in predicting the TG response to Fb, and a fourth showed that inclusion of SNP–CpG interactions improves the prediction of posttreatment TG levels.

In the first study, Cantor et al. [2] addressed the very fundamental question of whether any CpG sites are responsive to Fb. Their study design used a novel approach to address the confounding batch effect between pretreatment and posttreatment ML. They searched for those ML reflecting a posttreatment genetic contribution by filtering the posttreatment familiality and variability of ML distributions for outliers. Increased familiality and variability are hallmarks of a genetic effect [19]. Two genes, *ANAPC2* and *KIAA1804*, were selected, and both also had highly significant meQTL, providing support for the existence of Fb-responsive CpG sites. In the second, Wu et al. [5] conducted a very broad genome-wide investigation of *cis*-meQTL. By using LMM, they identified 229 SNPs associated with ML changes at 610 CpG sites. Among those, there were several consistent with what was reported previously. The most significant, located upstream of *MGAT1*, is known to be related to TG levels or lipid accumulation [20]. Enrichment analysis using the National Human Genome Research Institute genome-wide association studies (GWAS) catalogue identified 6 SNPs colocalized with 8 previously documented disease loci. Site cg09222892, located in gene *RHCE*, is associated with a well-known lipid SNP, rs10903129, in the gene *TMEM57*. These studies provide additional support for the existence of Fb-responsive CpG sites.

In the third study, Xia et al. [6] evaluated the contribution of ML in predicting a 30% reduction in TG levels using stratified risk modeling and ANN. Including ML in their models reduced the error rate by 4%, indicating that methylation data contributes to prediction accuracy of the drug response. The top predictors, rs10521308 (*FTO*), rs2206135 (*CTNNBL1*), cg13438334 (*DGAT1*), and cg22390041 (*ALDH4A1)* are located in genes known to be associated with obesity risk. In the fourth study, Yasmeen et al. [9] used simulated posttreatment TG levels to evaluate KST models for identifying associated regions around 5 causal and 5 noncausal CpG sites. Models without SNP–ML interactions were nonsignificant; however, when these interactions were included, significant *p* values were observed. Their results support the importance of considering the interactions of SNPs and ML when modeling the effects of Fb on TG levels, and illustrate that KST is appropriate for modeling treatment response with epigenetic data.

### Support for common variants in TG-level responses to fb

Two manuscripts provided support for the association of common variants with the TG-level response to Fb. Xu et al. [7] identify plausible SNP associations using LMM. Their top SNP, rs964184, is associated with lipid-lowering statin treatment [21]. Gene-based rare variant association testing revealed 6 meeting false discovery rate criteria. In addition, *DNMT3L*, which is known to regulate DNA methylation activity and is associated with obesity [22], was identified. Yang and Chen [8] conducted a more complex analysis to identify SNPs associated with TG levels. This study investigated homozygosity disequilibrium by identifying nonrandom patterns of homozygosity using homozygous intensity scores, GEE, and a sliding window. This phenomenon has been implicated in both Mendelian and complex diseases. Three regions surrounding rs254239, rs7037978, and rs17704829 provide support for the importance of *MACROD2* in the response to Fb.

### Analytic and study design considerations in predicting the response to fb

Two papers focused on analytic questions regarding sample size and correction for the nonindependence of pedigree members. Cherlin et al. [3] explored the predictive ability for drug response by penalized regression methods, providing evidence that a large sample size is needed to achieve good predictions. GWAS using LASSO regression on 680 individuals was conducted on posttreatment TG levels in the simulated and real data with pretreatment TG levels as the baseline, resulting in poor prediction. An analysis of a much larger independent data set showed a much better prediction with the same method, suggesting that a sample size of a few thousand individuals is needed to achieve good prediction with LASSO. In the second paper, Hsu et al. [4] evaluated the effect of adjusting for family structure. As expected, only analyzing unrelated subjects, consisting of 1 representative from each family, reduced power substantially, compared to using LMM or treating the pedigree members as independent. These manuscripts provide support for collecting a large sample and using the full sample when analyzing pedigrees.

## Discussion

This manuscript summarizes the aims, methods, study designs and results of 8 GAW20 investigations that were grouped together because they focused on the genetics of responses to treatment with Fb and the methods to examine it. The questions addressed and methods applied were derived from the longitudinal TG and ML data collected before and after Fb treatment in the GOLDN study. SNP data permitted both targeted and genome-wide assessments of genetic associations with TGs. ML data on CpG sites permitted targeted and genome-wide meQTL analyses. Genetics and genomics data on the same individuals undergoing treatment allowed an analysis of their interactions in the prediction of response. Analytic methods, where the number of genetic and genomic predictors is larger than the sample size, were applied, and findings indicate that an adequate sample size is critical. The 8 manuscripts clearly illustrate that correcting for the nonindependence of individuals within pedigrees using LMM to identify SNP associations with Fb response is straightforward, but correction when developing more complex models is not. This suggests that there is a need for the development of additional methods to accommodate such data.

The variability among individuals in their responses to drug treatment is often ignored, but as medicine moves toward greater precision in caring for patients, this area of investigation will grow. Currently, the complex influences on drug responses are understudied and often unknown, although there are exceptions for those variants exhibiting a Mendelian impact. The work summarized here clearly indicates that the inclusion of genetics and genomics data in a longitudinal drug treatment study is feasible and that such study has the potential to affect the precision of prediction.

The 8 summarized papers explore the genetic and genomic influences on the differences in drug responses among individuals and the appropriateness of study design elements and analytic methods to detect them. For example, the novelty of measuring pretreatment and posttreatment epigenetic ML invited questions regarding the responses of CpG sites to treatment with Fb, as well as the predictive role of ML changes in the known TG response to Fb. The former studies were hampered by a batch effect between pretreatment and posttreatment ML, and this confounding design element should provide a note of caution to future studies. However, these data and the analyses we report were successful in providing support for the notion that the ML of some CpG sites respond to Fb. Future studies can be designed to ensure there is no confounding batch effect, and the specific findings identified here can be studied for replication. In addition, given an unforeseen batch effect, a genetic approach to identify candidate CpG sites for meQTL studies, like the one used here [2], may be appropriate.

Study design remains an important issue for drug response work, and a critical issue is the development of adequate samples. As with other studies of complex traits, effect sizes of individual SNPs and CpG sites are likely to be small and difficult to detect. This is especially important when there is genome-wide multiple testing of interactions, and rare variants. In addition to sample size, the nonindependence of pedigree members was a concern. One may posit that the number of samples in the GOLDN pedigrees would provide more statistical power if they were collected on independent individuals. However, family data are likely to be more homogeneous, which can increase statistical power. The studies reported here corrected for the nonindependence of the pedigree members rather than capitalizing on the genetic transmission of information among family members. Although studying the transmission of the drug response in pedigrees is a more attractive approach, having complete data is unlikely because only some pedigree members take the treatment drug.

## Conclusions

Several conclusions are drawable from the 8 GAW20 manuscripts addressing responses to treatment with Fb summarized here. Regarding genomics, we can conclude that some CpG ML are responsive to Fb. In addition, CpG ML should be included in models predicting the TG responses to Fb. Regarding genetic contributions, both common and rare variants are associated with TG responses to Fb. Furthermore, genetics and genomics should be combined to include the interactions of common variants and CpG ML in models predicting the TG level response to Fb. Regarding study designs, multiple classes of models and statistical analyses are appropriate for these studies, and sample size is a critical factor in the successful construction of predictive models representing the response to Fb.

## Abbreviations

ANCOVA: Analysis of covariance
ANN: Artificial neural network
CpG: Cytosine-phosphate-guanine
Fb: Fenofibrate
GAW20: Genetic Analysis Workshop 20
GEEs: Generalized estimating equations
GOLDN: Genetics of Lipid Lowering Drugs and Diet Network
GWAS: Genome-wide association studies
KST: Kernel score test
LASSO: Least absolute shrinkage and selection operator
LMMs: Linear mixed models
meQTL: Methylation quantitative trait locus
ML: Methylation levels
PCs: Principal components
postTG: Posttreatment TG level
preTG: Pretreatment TG level
SNPs: Single-nucleotide polymorphisms
TG: Triglyceride
TG1, TG2, TG3 and TG4: TG measured at times 1,2,3 and 4
